# Enhancing Multispectral Breast Imaging Quality Through Frame Accumulation and Hybrid GA-CPSO Registration

**DOI:** 10.3390/bioengineering11121281

**Published:** 2024-12-17

**Authors:** Tsabeeh Salah M. Mahmoud, Adnan Munawar, Muhammad Zeeshan Nawaz, Yuanyuan Chen

**Affiliations:** 1 Medical School, Faculty of Medicine, Tianjin University, Tianjin 300072, China; tsabeeh.salah@tju.edu.cn (T.S.M.M.); adnanmunawar@tju.edu.cn (A.M.); zeeshannawaz@tju.edu.cn (M.Z.N.); 2 State Key Laboratory of Advanced Medical Materials and Devices, Tianjin University, Tianjin 300072, China; 3 Haihe Laboratory of Brain-Computer Interaction and Human-Machine Integration, Tianjin 300072, China; 4 Tianjin Key Laboratory of Brain Science and Neuroengineering, Tianjin 300072, China

**Keywords:** breast cancer, multispectral transmission imaging, frame accumulation, image registration, constriction factor, particle swarm optimization, genetic algorithm

## Abstract

Multispectral transmission imaging has emerged as a promising technique for imaging breast tissue with high resolution. However, the method encounters challenges such as low grayscale, noisy transmission images with weak signals, primarily due to the strong absorption and scattering of light in breast tissue. A common approach to improve the signal-to-noise ratio (SNR) and overall image quality is frame accumulation. However, factors such as camera jitter and respiratory motion during image acquisition can cause frame misalignment, degrading the quality of the accumulated image. To address these issues, this study proposes a novel image registration method. A hybrid approach combining a genetic algorithm (GA) and a constriction factor-based particle swarm optimization (CPSO), referred to as GA-CPSO, is applied for image registration before frame accumulation. The efficiency of this hybrid method is enhanced by incorporating a squared constriction factor (SCF), which speeds up the registration process and improves convergence towards optimal solutions. The GA identifies potential solutions, which are then refined by CPSO to expedite convergence. This methodology was validated on the sequence of breast frames taken at 600 nm, 620 nm, 670 nm, and 760 nm wavelength of light and proved the enhancement of accuracy by various mathematical assessments. It demonstrated high accuracy (99.93%) and reduced registration time. As a result, the GA-CPSO approach significantly improves the effectiveness of frame accumulation and enhances overall image quality. This study explored the groundwork for precise multispectral transmission image segmentation and classification.

## 1. Introduction

Breast cancer is a major global health issue, and is the most common cancer worldwide across various regions and socioeconomic backgrounds [1,2]. In 2020, it accounted for approximately 2.3 million new cases and 685,000 deaths, emphasizing its widespread impact [1]. Early detection is crucial for reducing mortality rates and improving treatment outcomes, underscoring the ongoing need for advancements in screening and prevention [3,4]. Current breast cancer screening methods include mammography [5], ultrasound [6], magnetic resonance imaging (MRI), and clinical breast exams [7,8]. While mammography is common, concerns about ionizing radiation persist [5]. Ultrasound may miss early-stage cancers, particularly in dense breast tissue [9], and MRI, though sensitive, is costly [10]. Clinical breast exams may also fail to detect early indicators. Thus, safer and more effective screening techniques are urgently needed for early detection.

Optical imaging has emerged as a promising alternative, using visible and near-infrared light for possible early breast cancer detection. It is cost-effective, free of radiation, and potentially suitable for home use [11,12].

Since Cheatle’s study of breast optical properties in 1929 [13], researchers have been working towards developing optical techniques that may contribute to the early diagnosis of breast diseases. The initial efforts focused on transmission imaging [14,15], which sought to detect breast abnormalities by visualizing lumps and vascular structures but often faced issues with accuracy. Recently, multispectral imaging has gained considerable attention as a potential solution to the limitations of traditional transmission imaging. Given that biological tissues have various components with unique absorption spectra, multispectral light passing through these tissues can produce monochromatic spectral images that reflect their absorption characteristics. This approach leverages the different absorption and scattering properties of tissues at multiple wavelengths, offering more detailed information that aids in identifying lesions [16]. As a result, multispectral transmission imaging—an integration of transmission and multispectral techniques—has become a valuable area of preclinical studies that may complement existing imaging techniques for early breast cancer screening [17].

However, challenges arise in breast tissue due to strong scattering and absorption characteristics, resulting in low grayscale, noisy transmission images with weak signals.

Low signal intensity affects the signal-to-noise ratio (SNR) and image quality adversely. Frame accumulation is widely used to improve the low SNR and enhance grayscale resolution [18]. Specifically, this approach integrates a given number of image frames by summing up the corresponding pixel values. The resultant image contains the accumulated signal with improved SNR [19,20]. However, the effectiveness of the frame accumulation technique in enhancing image quality may be compromised due to relative displacements between individual image frames [21,22]. The potential sources for such displacements between successive image frames include camera jitter and respiratory motion, particularly for tissue where the respiratory movement is more pronounced (e.g., breast tissues) [23]. The presence of relative displacements significantly hampers the effectiveness of SNR improvement through frame accumulation.

Image registration is an image processing technique designed to rectify relative frame displacement and enhance the accuracy of frame accumulation [24,25]. The process of image registration initiates with extracting features, such as the signal intensity of each pixel, from individual image frames. Subsequently, a suitable transformation model is selected for feature matching of different frames [26,27]. To align multiple frames with a standard reference frame [28,29], transformative operations may include corrections for changes in scale, rotation, translation, and even deformations [30,31]. Optimization techniques, such as gradient descent [32], Powell’s method [33], particle swarm optimization (PSO) [34], genetic algorithms (GAs) [35], and deep learning-based approaches [36,37], are employed to iteratively adjust transformation parameters for optimal frame alignment [38]. Finally, a cost function, acting as a similarity metric, is implemented to quantify the similarity between reference and moving images. Commonly used cost functions encompass mean squared error (MSE) [39], mutual information (MI) [40], and normalized cross-correlation (NCC) [41,42]. The frame alignment achieved through image registration facilitates accurate comparison, analysis, and combination of images.

The statement of the problem for this study was to address the displacement of image frames during sequential acquisition, which arises from respiration-associated movements and camera jitter. These displacements compromise the accuracy of frame accumulation and subsequently degrade image quality. In this study, a novel technique to improve the accuracy of image registration during frame accumulation was employed. Briefly, a hybrid approach integrated the use of GAs and CPSO algorithms for image registration. The strong global search capabilities of GAs provided the coarse refinement, while the CPSO algorithm was effective in fine-tuning. By combining these two algorithms, the hybrid GA-CPSO approach enhanced the accuracy of the image registration process. Additionally, a squared constriction factor (SCF) was introduced in the CPSO algorithm, referred to as the SCF-based CPSO algorithm, which increased the speed of registration. Validation of the approach was performed using multispectral breast images at four wavelengths: 600 nm, 620 nm, 670 nm, and 760 nm. The results demonstrated a significant improvement in registration accuracy and time efficiency, evaluated through multiple metrics and compared with other methods. The results demonstrated that the GA-CPSO technique improved the accuracy of frame accumulation for sequentially acquired images, leading to measurable enhancements in image quality, indicated by improvements in image quality metrics such as contrast, edge sharpness, and focus.

## 2. Theories and Methods

### 2.1. Squared Constriction Factor-Based Particle Swarm Optimization (CPSO) Algorithm

The PSO involves a collection of entities referred to as a swarm, with each member termed a particle. Visualized as points within the search space, particles aim to converge towards positions where optimized results are discerned [43]. In the pursuit of PSO, optimal outcomes of individual particles are adapted by considering their prior experiences and interactions with neighboring particles. This study used the PSO algorithm due to its effectiveness, simplicity, ease of implementation, applicability to diverse problems, and strong global optimization capabilities, all of which contribute to enhance image quality and registration [44]. The formulation of the PSO algorithm can be expressed as:

(1) $$v_{i}^{t+1}=w^{t}v_{i}^{t}+c_{1}\cdot \;\mathrm{rand}\;\cdot \;\left[p\_{\mathrm{best}\;}_{i}-x_{i}^{t}\right]\;+c_{2}\cdot rand\cdot \left[g_{\mathrm{best}\;}-x_{i}^{t}\right]$$

(2) $$w^{t+1}=w^{t}+dw;\;dw=\frac{\left(w_{min}-w_{max}\right)}{T}$$

(3) $$x_{i}^{t+1}=x_{i}^{t}+v_{i}^{t+1}$$

During iteration t, $x_{i}\;$ denotes the ith particle moving with a velocity vector $v_{i}$, $p\_{best}_{i}\;$ refers to the personal best of $x_{i}$, and $\;g_{best}$ represents the global best among all particles. The weight w, along with acceleration constants $c_{1}$ and $c_{2}$, influences the particle’s movement. The term “rand” corresponds to a random number uniformly distributed between 0 to 1 [45].

During image registration in PSO, the particle x signifies the transform parameter underestimation. The $p\_{best}_{i}\;$ corresponds to the minimum MSE (i.e., the cost function) for each particle [46,47]. The determination of $g_{best}$ is based on the cluster MSE.

A constriction factor is introduced to regulate the particle velocities throughout the optimization process, which encourages a balance between exploration and exploitation. The constriction factor imposes a limit on the maximum particle velocity to ensure convergence toward the best solution while preventing excessive particle movement.

The conventional constriction factor (χ) is calculated using the acceleration constants $c_{1}$ and $c_{2}$ to update the velocity, such that

(4) $$\chi =\frac{2}{\left|2-\emptyset -\sqrt{\emptyset^{2}-4\emptyset }\right|},\;\;where\;\emptyset =c_{1}+c_{2},\;\emptyset >4$$

The velocity equation of the ith particle with χ can be calculated as

(5) $$\begin{array}{rr} v_{i}^{t+1}= & {\chi [w}^{t}v_{i}^{t}+c_{1}\cdot \;\mathrm{rand}\;\cdot \left[p_{{\mathrm{best}\;}_{i}}-x_{i}^{t}\right]+c_{2}\cdot rand\cdot \left[g_{\mathrm{best}\;}-x_{i}^{t}\right]] \end{array}$$

A novel approach of this study was introducing a squared constriction factor $\left(\chi^{2}\right)$ to the PSO algorithm (SCF-based CPSO). This approach amplified the benefits of the conventional constriction factor (CCF)-based CPSO. Specifically, using SCF, our approach effectively emphasized the influence of the regularization mechanism, contributing to improved convergence dynamics.

The SCF-based CPSO algorithm exhibits commendable local optimization capabilities. However, when the initial particle positions are randomly selected, it not only prolongs the early-stage search process in CPSO but also raises the risk of getting trapped in local extremes. To address this challenge, we propose the integration of a genetic algorithm (GA) to serve as the initial point for CPSO. By utilizing the best solutions obtained from the GA to initialize CPSO particles, we aimed to mitigate the disadvantages of random initialization, enhancing the efficiency of the optimization process and minimizing the likelihood of converging to local extremums.

### 2.2. Genetic Algorithm

Gas are a widely used optimization technique based on natural selection and genetic mechanisms [48,49]. GAs use an evolutionary process to find optimal solutions for intricate problems. They initialize with a population of potential solutions, each represented as individuals (chromosomes). Over successive generations, these individuals undergo processes reminiscent of natural selection, crossover, and mutation [48,50].

In image registration, the selection phase hinges on choosing individuals based on their fitness, reflecting their efficacy in addressing the registration task. Crossover strategically combines genetic information from two parent solutions to produce offspring, while mutation introduces subtle random variations. This cyclic process persists, with fitter individuals being more likely to be chosen, ultimately driving the algorithm toward optimal or near-optimal solutions. GAs are particularly suitable for image registration applications because of their capacity to explore a wide solution space.

The GA employed in this study consisted of the following parameters:

- A population size of 50 chromosomes;
- The Roulette wheel method for selection of individual reproduction;
- The mean squared error (MSE) as fitness evaluation function;
- A one-point crossover operation with a probability of 0.8;
- Introduction of Gaussian noise (mimicking mutation process) with a probability of 0.05;
- Execution of GA for 50 generations (i.e., iterations) to identify optimal solution space.

### 2.3. Cost Function (Similarity Metric)

The objective was to use the hybrid GA-CPSO algorithm to effectively register and align the moving image with the fixed image, optimizing the cost function used in this study, which was the mean squared error (MSE). The cost function evaluated the alignment of the fixed and moving images during image registration. MSE is a widely used similarity metric in image registration due to its simplicity and effectiveness in quantifying image similarity [51]. MSE quantified the similarity between two images by calculating the average squared differences of pixel intensities at corresponding locations, as given by

(6) $$MSE\left(I_{F},I_{M}\right)=\frac{1}{n}\;\sum_{i=1}^{n}{\left(I_{F(i)}-I_{M}(T_{\left(i,p\right)})\right)}^{2}$$

where $I_{F}\;$ and $I_{M}$ are representations of the fixed image and moving image, respectively, the variable ‘n’ signifies the total pixel count in the image. ‘T’ denotes the transformation applied to the moving image using two-dimensional parameters p ($t_{x}$, $t_{y}$, θ) before proceeding with the MSE calculation. The transformation parameters include translation in the x and y directions, as well as the rotation angle θ.

The translation parameters and the rotation angle were initially set to zero ($t_{x}=0$, $t_{y}=0$, θ = 0). However, these parameters were continuously updated throughout the process to achieve the best alignment between the two images during the execution of the hybrid GA-CPSO algorithm.

In this process, the GA first conducted a global search to identify optimal regions within the solution space by simulating evolutionary processes, treating the translation and rotation parameters as individuals in a population. The best values identified by the GA then served as the initial swarm positions for the CPSO phase. This strategic approach ensured that the CPSO starts with a refined set of parameters, enhancing its efficiency in fine-tuning the alignment. CPSO leveraged the social behavior of particles, adjusting the translation and rotation parameters ($t_{x}$, $\;t_{y}$, θ) towards the most promising solutions based on both individual and collective achievements. By integrating the global search capabilities of GAs with the precise optimization of CPSO, the algorithm ensured a seamless and progressively accurate adjustment of the transformation parameters for optimal image alignment.

**Figure 1:**
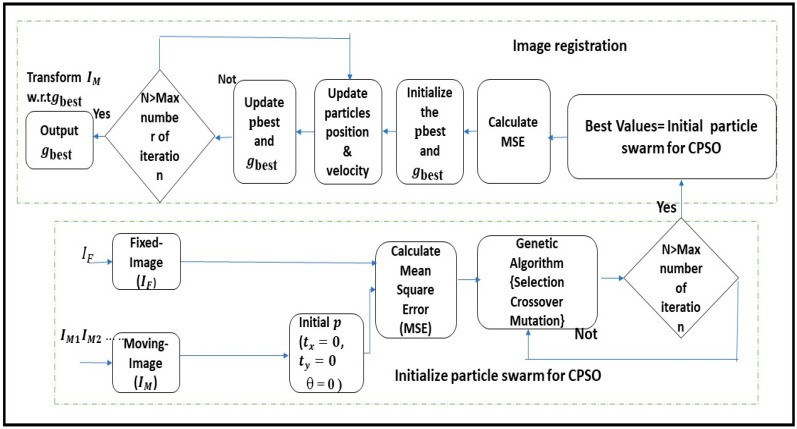
Flow chart of GA-CPSO registration algorithm.

Figure 1 presents a schematic of the GA-CPSO algorithm for image registration. Briefly, the algorithm initialized by assuming image transformation parameters between the fixed image ($I_{F}$) and the moving image ($I_{M}$) at zero (i.e., $t_{x}=0$, $t_{y}=0$, θ = 0). In the first phase (i.e., execution of GA), random values were generated for the population of potential solutions, followed by MSE calculation between $I_{F}$ and $I_{M}$ for each individual in the population. Algorithm iteration continuously improved (i.e., reduced MSE) the population through selection, crossover, and mutation operations, until either the maximum number of generations or convergence of optimization. At this stage, the final values of transformation parameters were obtained, which served as input for the second phase of the algorithm (i.e., execution of CPSO), where the initial positions and velocities of the particles were adjusted. Afterwards, the MSE was recalculated to iteratively update the particles’ positions until the personal best ($p_{\mathrm{best}\;}$) and global best ($g_{\mathrm{best}\;}$) were achieved. The SCF ensured precise convergence to the global optimum. The process repeated until the CPSO phase reached the maximum iteration, outputting the final optimized parameters ($t_{x}$, $t_{y}$, θ), which were applied to transform the moving image for precise alignment with the fixed image.

### 2.4. Principles of Frame Accumulation

Breast multispectral transmission imaging often yields a weak SNR with low grayscale due to the turbid nature of biological tissues. Frame accumulation is a promising technique to enhance such weak signals effectively. Currently, there are two primary approaches to achieve frame accumulation: superimposing multiple frame images and extending the frame integration time. While extending the frame integration time can increase signal strength, it often leads to issues such as image overexposure or blurring, thereby compromising image quality. Consequently, in this study, we adopted the method of accumulating multiple frame images captured at different times to improve the overall image quality by enhancing signal strength and reducing noise.

If $x_{s}$ represents the ideal signal in the image, and $x_{n}$ represents the random noise carried in the image, then the image SNR is:

(7) $${SNR}_{1}=\frac{x_{s}}{\sigma }$$

where σ represents the standard deviation of the random noise. After frame accumulation of *m* frames of images, the standard deviation of the noise within the signal is reduced to */*$\sqrt{m}$. Consequently, the SNR of the image can be calculated as follows:

(8) $${SNR}_{m}=\frac{\sum_{i=1}^{m}x_{si}}{\sum_{i=1}^{m}\sigma /\sqrt{m}}=\sqrt{m}{SNR}_{1}$$

This formula indicates that through the process of frame accumulation, the SNR of the image is effectively increased by a factor of $\sqrt{m}$.

### 2.5. Evaluation Metrics

The accuracy of the proposed image registration method was evaluated using several metrics, including correlation coefficient (CC), MI, root mean square error (RMSE), and registration accuracy.

The CC is a measure of the linear relationship between pixel intensities between two images [52], given as

(9) $$CC=\frac{\sum_{x=1}^{m}\sum_{y=1}^{n}\left[{(I}_{F\left(x,y\right)}-\bar{I_{F}}\right){(I}_{Reg\left(x,y\right)}-\bar{I_{Reg}})]}{\sqrt{\left[\sum_{x=1}^{m}\sum_{y=1}^{n}{{(I}_{F\left(x,y\right)}-\bar{I_{F}})}^{2}\times \sum_{x=1}^{m}\sum_{y=1}^{n}{{(I}_{Reg\left(x,y\right)}-\bar{I_{Reg}})}^{2}\right]}}$$

A value of 1 indicates a perfect positive linear correlation (i.e., identical images), a −1 implies a perfect negative correlation, while 0 indicates no linear correlation.

The MI measures the statistical dependence between the intensity distributions of two images, signifying the information each image provides about the other.

(10) $$MI=\sum_{x=1}^{m}\sum_{y=1}^{n}P{(I}_{F(x,y)},\;I_{Reg(x,y)})\times og\;\left(\frac{P\left(I_{F\left(x,y\right)},I_{Reg\left(x,y\right)}\right)}{P\left(I_{F\left(x,y\right)}\right)\times P(I_{Reg\left(x,y\right)})}\right)$$

In image registration, maximizing MI aids in identifying the optimal alignment by evaluating the deviation of their joint intensity distribution from the product of their distributions [53].

The root mean square error (RMSE) calculates the average magnitude of pixel-wise differences between the registered and reference images, offering a measure of overall dissimilarity. Mathematically,

(11) $$RMSE=\sqrt{\frac{1}{m\times n}\sum_{x=1}^{m}\sum_{y=1}^{n}{{(I}_{F}\left(x,y\right)-I_{Reg}(x,y))}^{2}}$$

Smaller RMSE values indicate superior alignment, signifying close matching of pixel intensity between the registered and reference images.

Accuracy, derived from the set of moving and alignment parameters, is computed as

(12) $$A=\frac{1}{3}\left(\frac{\left|X-x\right|}{X}+\frac{\left|Y-y\right|}{Y}+\frac{\left|R-r\right|}{R}\right)\times 100\%$$

where x and y  are the pixel intensities at corresponding positions in the fixed image ${(I}_{F}$) and registered image ${(I}_{Reg}$), while m and n show the number of rows and columns, respectively. $\bar{I_{F}}$ shows the average of the intensity values of fixed image ${(I}_{F}$), and $\bar{I_{Reg}}$ shows the average of the intensity values of the registered image ${(I}_{Reg})$. $P\left(I_{F\left(x,y\right)}\right)$ is the probability distribution of pixel intensities in the image $I_{F}$. $P(I_{Reg\left(x,y\right)})$ is the probability distribution of pixel intensities in the image $I_{Reg\left(x,y\right)}$. $P(I_{F\left(x,y\right)},I_{Reg(x,y)})$ is the joint probability distribution of pixel intensities in images $I_{F}$ and $I_{Reg}$.

The following metrics were used to evaluate the image quality after applying the registration and accumulation techniques.

The gray level (GL) characterizes the grayscale resolution of the image, signifying its informational content, with higher values indicating increased contrast [52].

The standard deviation (SD) quantifies the degree of pixel intensity variation, offering insights into image clarity, as given by

(13) $$SD=\sqrt{\frac{1}{m\times n}\sum_{x=1}^{m}\sum_{y=1}^{n}(f(x,y)-\mu {)}^{2}},\;\mathrm{where}\;\mu =\frac{1}{m\times n}\sum_{x=1}^{m}\sum_{y=1}^{n}f(x,y)$$

where m and n represent the total number of rows and columns in the image, and *f*(*x*,*y*) denotes the gray value at the xth row and yth column.

Entropy quantifies the randomness and complexity of pixel distribution, providing information about image texture:

(14) $$\;\mathrm{Entropy}\;=-\sum_{x=0}^{g}P(x){log}_{2}P(x)$$

where *P*(*x*) signifies the probability of a pixel having the value x in the image, and g is the range of gray values.

The energy of image gradient (EOG) represents the energy of image gradients, offering insights into the image’s sharpness and structural details as [52]

(15) $$EOG=\sum_{x=1}^{m}\sum_{y=1}^{n}\left[(f\left(x+1,y\right)-{f(x,y))}^{2}+(f\left(x,y+1\right)-{f(x,y))}^{2}\right]$$

The Brenner gradient function (BGF) measures image sharpness by analyzing intensity variations between adjacent pixels as follows [54]

(16) $$BGF=\sum_{x=1}^{m}\sum_{y=1}^{n}{\left[f\left(x+2,y\right)-f(x,y)\right]}^{2}$$

The multiple of the modulus of gray difference (MMD) quantifies the sharpness and contrast of an image, offering insights into the image’s clarity and feature distinction as:

(17) $$MMD=\sum_{x=1}^{m}\sum_{y=1}^{n}|f(x,y)-f(x+1,y)|*|f(x,y)-f(x,y+1)|$$

## 3. Experimental Validation

To assess the efficacy of the proposed approach, we developed a multispectral breast transmission imaging system. The imaging setup was developed for pre-clinical evaluation and may not meet the standardization requirements for clinical diagnostic imaging. The focus was on demonstrating the technical feasibility of the GA-CPSO algorithm in improving image quality under experimental conditions. This section delineates the experimental setup, outlines the image acquisition process, and elaborates on the subsequent image processing steps.

### 3.1. Experimental Setup

The experimental setup comprised three primary components: an illumination system, an image acquisition module, and an image processing unit. The optical illumination for breast tissues consisted of four different LED systems, each emitting light at specific wavelengths (i.e., 600 nm, 620 nm, 670 nm, and 760 nm). This selection of optical wavelengths was informed by the light absorption properties of tumor tissues and the sensitivity range of the mobile phone camera, ensuring optimal detection capabilities. Moreover, for each wavelength, the LED system comprised 64 LEDs arranged in an 8 × 8 square matrix (all the LEDs in this array were designed to emit light at the same wavelength). This system was powered by portable lithium batteries, the highest current is 2.8 A; for the 600 nm and 620 nm LEDs the voltage supplied is 21 V, while for the 670 nm and 760 nm LEDs it is 14.8 V. An iPhone 12 Pro-Max rear camera was used to record videos of the human breast. The image processing was performed with an Acer desktop PC (Model Shangqi X4270) equipped with MATLAB version 2022a and Python 3.9.

**Figure 2:**
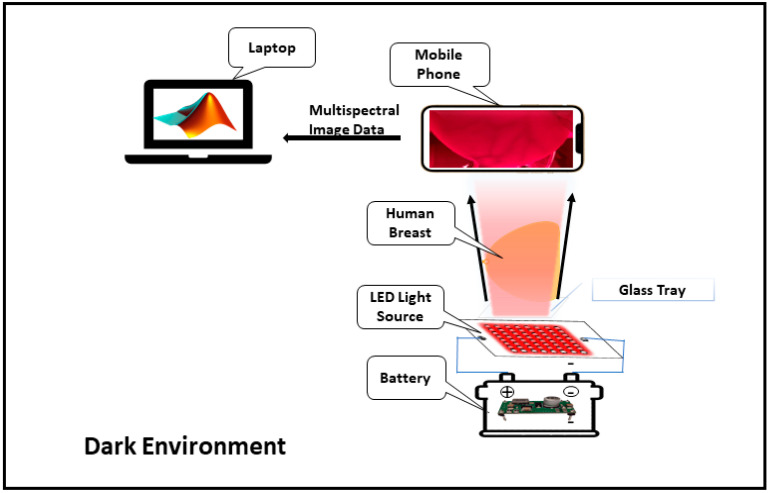
Schematic of the image acquisition system.

Figure 2 shows a schematic of the image acquisition process.

### 3.2. Image Acquisition

**Figure 3:**
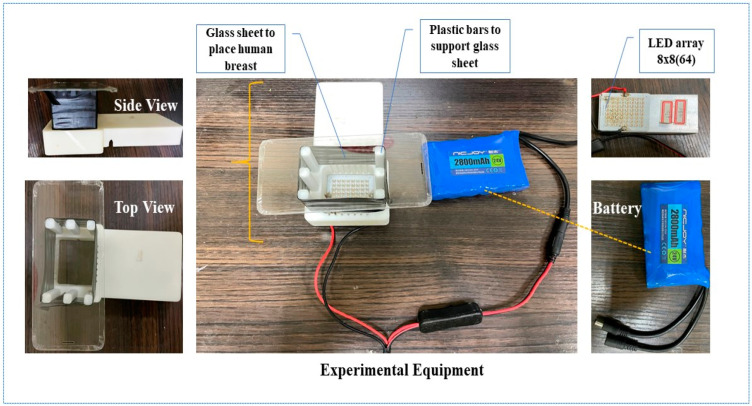
Experimental setup for multispectral image acquisition.

To initiate the experiment, the breast tissue was positioned to be directly illuminated by the light source while maintaining a 6 cm separation. The image acquisition unit was composed of a glass sheet supported by six plastic bars, each 6 cm in length, to ensure a consistent 6 cm gap between the light source and the breast. The plastic bars were wrapped in black tape to prevent any light leakage during the image acquisition process. The unit was placed over the LED light source to ensure controlled and consistent illumination. Additionally, the entire experiment was conducted in a fully dark room to avoid any external light interference. Figure 3 illustrates the experimental setup.

**Figure 4:**
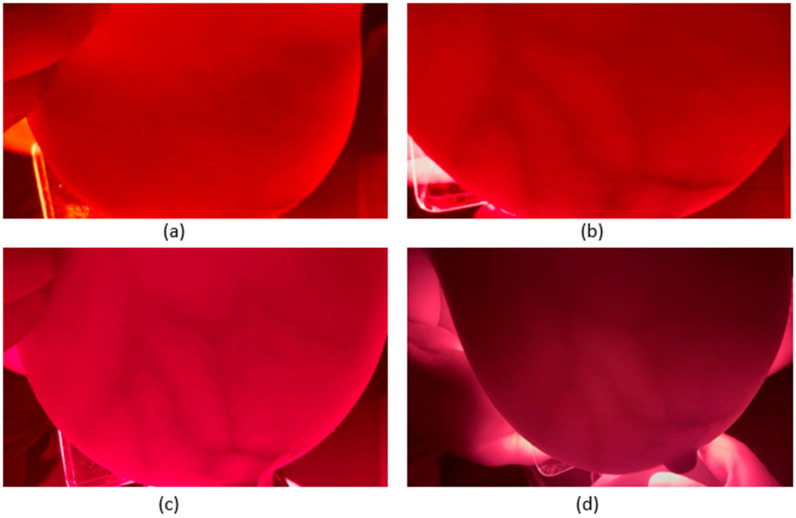
Illustrative multispectral transmission images of the human breast for Subject 1 captured at (**a**) 600 nm, (**b**) 620 nm, (**c**) 670 nm, and (**d**) 760 nm.

**Figure 5:**
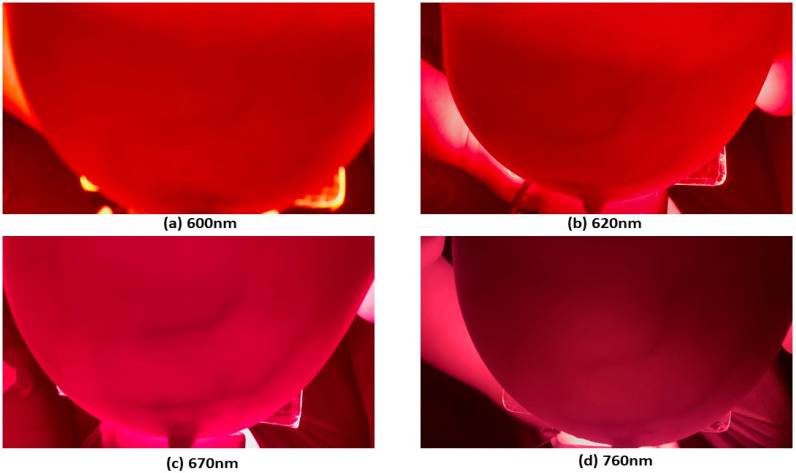
Illustrative multispectral transmission images of the human breast for Subject 2 captured at (**a**) 600 nm, (**b**) 620 nm, (**c**) 670 nm, and (**d**) 760 nm.

The handheld phone camera’s distance was adjusted and oriented to ensure complete breast tissue coverage and precise focus. It was ensured that the light was transmitted effectively without excessive camera glare. The data were acquired with the mobile phone camera by recording a 10 s video for each wavelength. This process was repeated for both subjects across all four wavelengths, resulting in a comprehensive dataset. The video frame rate was set to 60 frames per second (fps) for 600, 620, and 670 nm, and 30 fps for the 760 nm wavelength. This variation in frame rates was due to the camera’s automatic adjustment, with the brighter wavelengths (600 nm, 620 nm, and 670 nm) set to 60 fps and the darker 760 nm wavelength adjusted to 30 fps to optimize image quality. The first frame for each wavelength image is shown in Figure 4 and Figure 5 for both subjects, respectively.

#### Declaration

Volunteers were recruited as subjects, participating voluntarily after providing informed consent with full understanding of the study. All experiments were conducted in accordance with relevant ethical guidelines and the laws of the People’s Republic of China. The study followed the ethical standards of the Tianjin University ethics committee.

### 3.3. Image Processing

The processing of multispectral breast images will be illustrated using an example obtained with the 670 nm illumination from Subject 1. However, the described image processing steps—image pre-processing, multi-frame image registration, and frame accumulation—were applied consistently to the data collected, as detailed in Section 3.2, from both subjects across all wavelengths. A thorough explanation of the image processing methodology is provided in Section 3.3.1, Section 3.3.2 and Section 3.3.3

#### 3.3.1. Image Pre-Processing

Initially, 600 individual frames were extracted from the acquired video using Python software. Each extracted frame was in RGB format with a resolution of 3840 × 2160 pixels. Each frame was separated into distinct R, G, and B channels, resulting in $\;I_{R}$, $I_{G}$, and $I_{B}$. The average of these three channels for each frame resulted in the corresponding grayscale images.

We employed an image enhancement technique to address the low contrast in grayscale images. Specifically, a mean denoising filter was applied in all images to enhance the image quality. The denoised images were obtained by averaging pixel values in local 4 × 4 neighborhoods, effectively reducing image noise. This denoised image can be used for further processing, such as image registration. This process suppressed noise and enhanced the image.

#### 3.3.2. Multi-Frame Image Registration

Either adjacent frames or all frames can be registered concerning a single reference image. In this study, the latter approach was implemented by registering all other images (moving images) to the first frame image (referred to as the fixed image). The latter method reduces superposition errors between images. In particular, the hybrid GA-CPSO image registration process was implemented by selecting the first two frames, where the first frame was designated as the fixed image ($I_{F}$) while the second frame was denoted as the moving image ($I_{M}$).

1. The transformation process was initiated by applying initial input parameters ($t_{x}=0$, $t_{y}=0,\;\theta =0$) to the moving image.
2. The MSE between the fixed image and the transformed moving image was computed using Equation (6), which quantified the dissimilarity between the two images.
3. The GA and CPSO techniques were integrated for optimal image registration. The GA was executed for a specified number of generations (gaMaxGen), wherein each individual’s fitness is evaluated using the objective function. Roulette wheel selection was employed for the selection operation, and the population was updated accordingly. The one-point crossover was implemented with a specified probability, facilitating gene exchange beyond a randomly chosen crossover point. Additionally, mutation introduced Gaussian noise with a certain probability, promoting genetic diversity.

**Figure 6:**
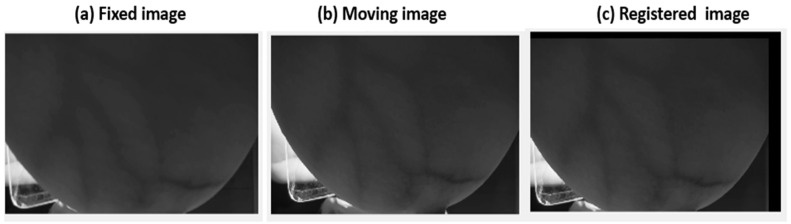
Image registration: fixed image (**a**), moving image (**b**), and registered image (**c**).

Following the GA’s optimization, the best values obtained (the value of $t_{x}$, $t_{y}$, and θ) were utilized to initialize particles in the CPSO phase. The constriction factor in CPSO was a critical parameter influencing particle movement in the optimization process. It was used to balance the exploration and exploitation phases. The constriction factor limited the particle’s velocity, preventing excessive movement and guiding the swarm toward optimal solutions. The proposed SCF in CPSO enhanced convergence speed and solution quality, offering improved optimization performance and stability in hybrid algorithms (Table 1). Specifically, tracking the best positions for each particle and the global best position ensured robust convergence. The “global best position” refers to the set of translation and rotation parameters ($t_{x}$, $\;t_{y}$, and θ) that result in the highest degree of alignment between the fixed and moving images, as determined by the lowest mean squared error (MSE). The final results were displayed, and the optimal translation and rotation was applied to register the moving image precisely with the fixed image, as depicted in Figure 6. The black edges in the registered image (c) indicate the shifts along the x and y axes, as well as the rotation, reflecting the changes in image alignment.

Table 1 highlights the significant advantages of the SCF in the CPSO algorithm compared to the CCF. The SCF-based CPSO achieved faster registration times, obtaining optimal values in early iterations and showcasing its efficiency in image registration. This notable difference underscores the enhanced algorithm’s superiority over CCF-based CPSO regarding both speed and effectiveness.

**Figure 7:**
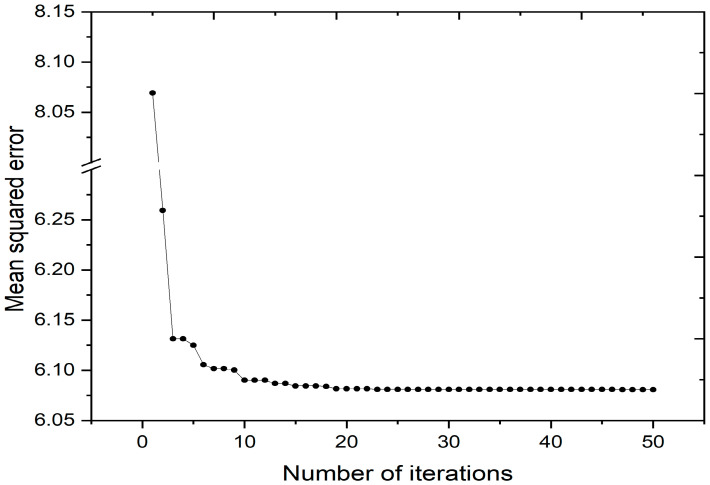
Optimization of the mean squared error.

Figure 7 visually depicts the MSE evolution throughout the registration process, initiating from an initial value of 8.069 and steadily converging to its minimum of 6.081 by the 47th iteration. The consistent and minimal fluctuations underscored the effectiveness of the GA-CPSO algorithm in mitigating premature convergence and oscillations around suboptimal solutions. By integrating a SCF in the CPSO algorithm, the enhanced approach achieved a delicate balance between exploration and exploitation, ensuring resilience against local optimal. The resulting transformation parameters at the minimum MSE value were (x = −1.4994, y = −0.5014, θ = 0.05) highlighting the algorithm’s capability to yield accurate and stable solutions during the registration process.

#### 3.3.3. Frame Accumulation

**Figure 8:**
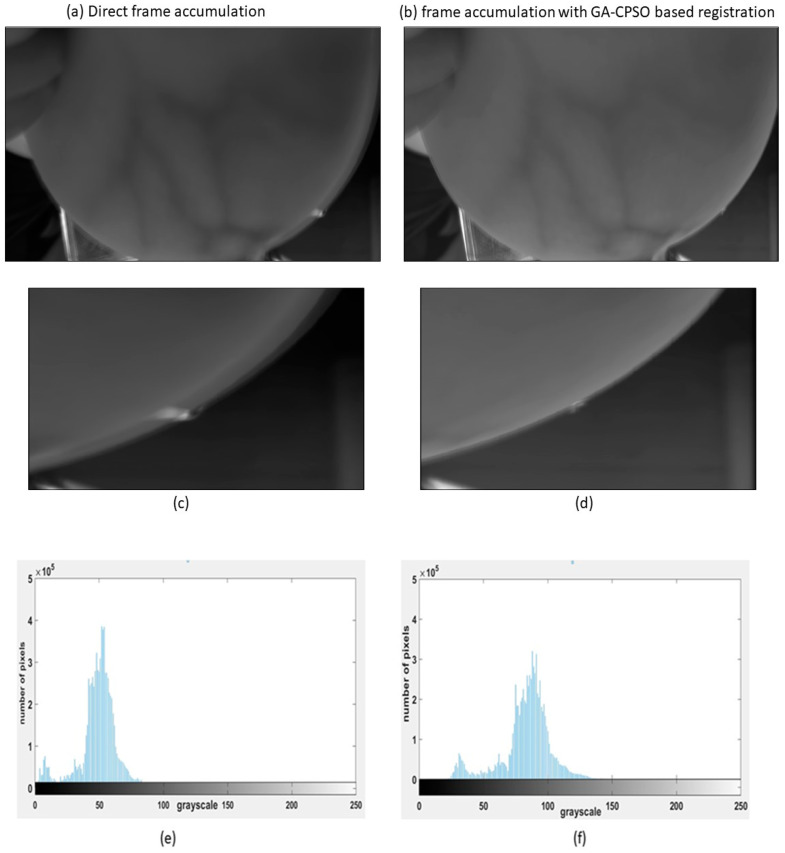
Frame accumulation comparison: (**a**) direct frame accumulation, and its corresponding histogram displayed in (**e**), (**b**) frame accumulation with GA-CPSO registration applied to 20 frames and its corresponding histogram shown in (**f**), (**c**) area cropped from the direct accumulation method presented in detail, (**d**) area cropped following GA-CPSO enhancement.

The novel registration method enhanced the alignment of the image sequence, which was further improved by accumulating 20 frames, as shown in Figure 8. By employing the GA-CPSO method for registration before accumulation, the approach significantly improved the signal-to-noise ratio and grayscale contrast of the images. Specifically, applying registration before accumulation improves image quality, especially noticeable in edge clarity where indicators are placed. To better illustrate the improved quality achieved by our method, we have selectively cropped the right areas of interest that show significant differences in Figure 8a,b. These regions are then magnified and displayed in Figure 8c,d, allowing for a clearer comparison of the enhancements.

## 4. Results and Discussion

This section evaluates the performance of the proposed hybrid GA-CPSO registration algorithm, demonstrating its significant improvements in registration accuracy and time efficiency compared to conventional methods. The method enhances the alignment of multispectral breast images. When combined with frame accumulation, it further improves overall image quality by increasing the signal-to-noise ratio, grayscale contrast, and edge clarity.

### 4.1. The Registration Method’s Performance Evaluation

To validate the efficiency of the novel registration algorithm proposed in this study, we conducted a thorough analysis of the registration outcomes using the set of moving images acquired with multispectral transmission of the breast tissue. To evaluate the similarity between the registered moving images and the reference image, the metrics employed were CC (Equation (9)), MI (Equation (10)), RMSE (Equation (11)), and accuracy (Equation (12)).

#### 4.1.1. Comparison of Image Registration: CPSO vs. GA-CPSO Algorithm

We began with a set of moving images and applied specific translational and rotational transformations to create transformed reference images. These transformations included shifts in both the horizontal and vertical directions, as well as rotations by predefined angles. The original moving images were then registered to these transformed reference images using both the CPSO algorithm and the hybrid GA-CPSO-based SCF algorithm. The goal was to evaluate how accurately each method could align the moving images with the transformed reference images. The results of the registration accuracy for both methods are summarized in Table 2 and Table 3.

**Figure 9:**
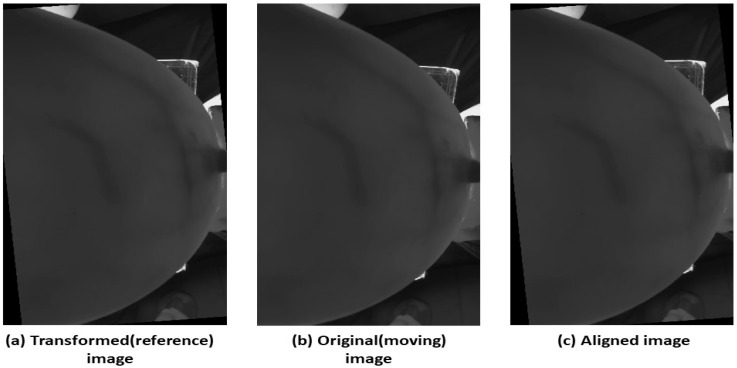
Example of Image Registration for Subject 2 at 670 nm: (**a**) transformed (reference) image with shift of (25, −20, 5), (**b**) original (moving) image, and (**c**) aligned image post-registration using GA-CPSO.

An example of this process is illustrated in Figure 9 for Subject 2 at 670 nm. The reference image was shifted by (25, −20, 5) and then the original (moving) image was registered to this transformed reference using the GA-CPSO algorithm, resulting in the aligned image.

Table 2 and Table 3 show that the hybrid GA-CPSO algorithm consistently outperformed CPSO alone for both subjects, achieving over 99% accuracy across all wavelengths. For Subject 1 (Table 2), the highest accuracy of 99.939% occurred with a shift of (50, −50, 10) at 670 nm. Similarly, Subject 2 (Table 3) showed a maximum accuracy of 99.935% at 760 nm with a shift of (−80, 60, 15). These results demonstrate the superior performance of GA-CPSO, particularly in handling large and small displacements, improving registration accuracy and image alignment across all cases.

#### 4.1.2. Comparison of Image Registration: GA-CPSO Algorithm vs. Other Registration Methods

The moving images captured at four different wavelengths (600, 620, 670, and 760 nm) were registered using three distinct techniques: the proposed hybrid GA-CPSO algorithm, the Powell algorithm [54], and the terrace compression method with normalized MI (TC-NMI) [55]. The results of these comparisons, as summarized in Table 4, are based on data from Subject 1. This analysis highlights the advantages of our method in terms of registration accuracy and registration time efficiency compared to the previous approaches.

For the GA-CPSO algorithm employed for images acquired at 600 nm, a robust linear relationship between the registered and fixed images was indicated by CC~0.9950, substantial shared information by MI~4.2474, and minimal differences RMSE~1.5129; these metrics were superior to the alternative methods of the Powell algorithm and the terrace compression. Similarly, for images acquired at 620, 670, and 760 nm, the GA-CPSO method consistently outperformed other methods in all evaluation metrics. Furthermore, our method demonstrated particularly superior performance in mean squared error (MSE), achieving the best value at 670 nm, which was 1.1218, in stark contrast to the TC-NMI method’s 15.1538, indicating the superior alignment capability of our method. Additionally, in the Powell algorithm at wavelength 620 nm, the MSE was 9.2852, and at 760 nm, it was 10.7126, while our method maintained the lowest values, 1.6419 and 2.9488, respectively, further substantiating the superior accuracy and efficiency of the hybrid GA-CPSO algorithm in image registration across all tested wavelengths.

Moreover, the GA-CPSO approach demonstrated substantially higher efficiency for registration time at all four wavelengths. The image registration time for the proposed GA-CPSO algorithm has been significantly reduced compared to the CCF-based PSO algorithm (Table 1) and other reported techniques (Table 4). Specifically, the image registration time for the GA-CPSO algorithm was an order of magnitude smaller than the Powell algorithm and TC-NMI algorithm, with times of 406.5 s for GA-CPSO compared to 3638.6 s and 3515.1 s, respectively. This trend was consistently observed for images acquired at all four wavelengths (600 nm, 620 nm, 670 nm, and 760 nm). Overall, these differences highlight the efficiency of the hybrid GA-CPSO method, making it an attractive choice for image registration with high precision and speed.

### 4.2. Evaluation of Frame Accumulation Performance After Registration

**Figure 10:**
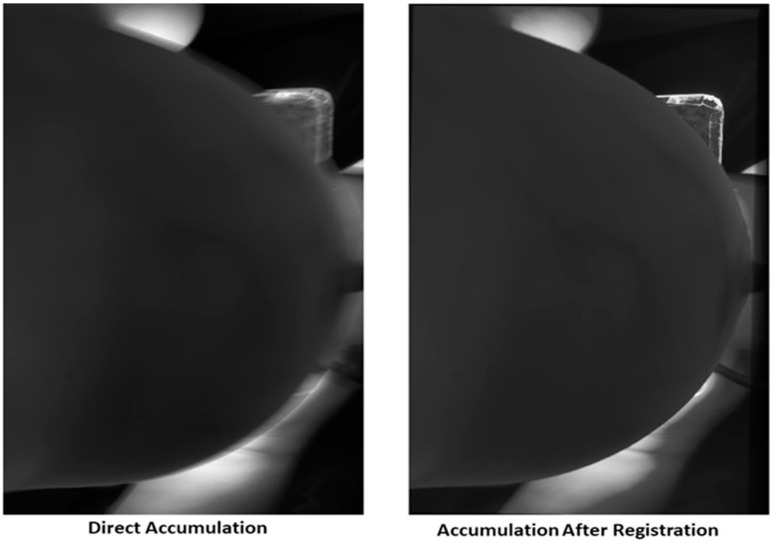
Comparison of image quality for direct accumulation and accumulation with registration for Subject 2 at 620 nm (20 Frames).

**Figure 11:**
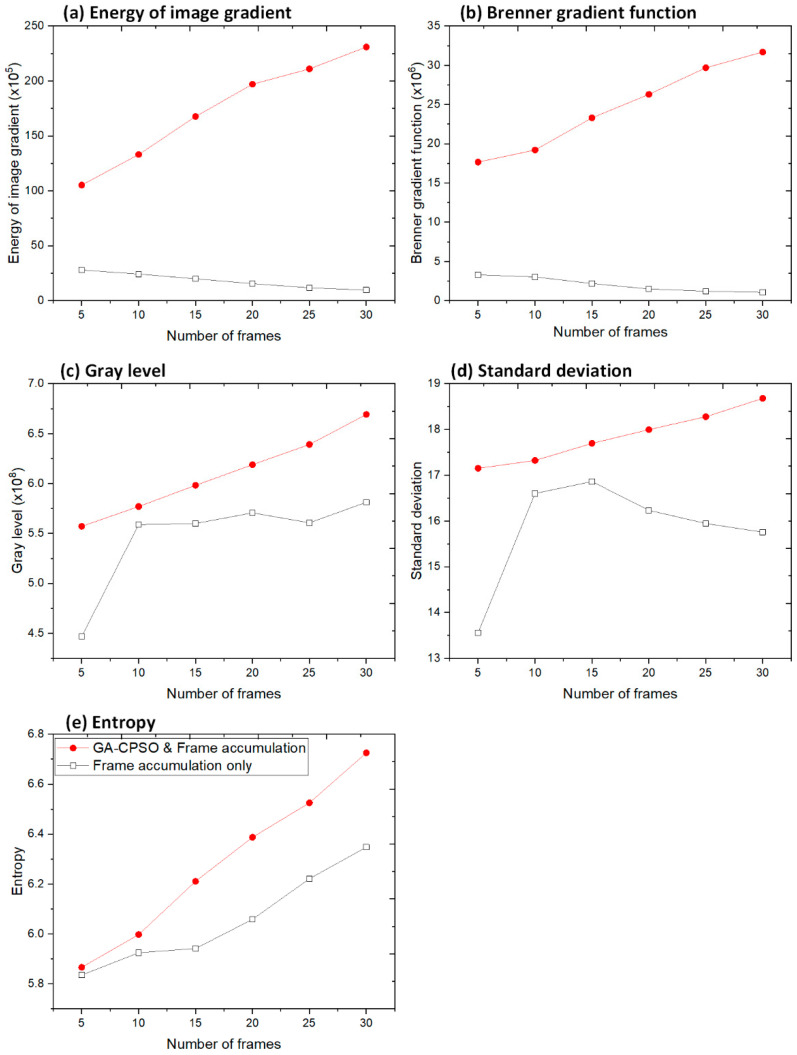
Comparison of image quality for frame accumulation only and GA-CPSO algorithm-based registration plus frame accumulation (Subject 1). The evaluation metrics include the energy of image gradient (**a**), Brenner gradient function (**b**), gray level (**c**), standard deviation (**d**), and entropy (**e**).

The impact of frame accumulation following registration was evaluated by comparing the image quality metrics for frame accumulation alone (i.e., without registration) and frame accumulation integrated with GA-CPSO-based registration. The results for Subject 1 are illustrated in Figure 10, showing how registration improves image quality based on metrics such as the energy of image gradient, Brenner gradient function, gray level, standard deviation, and entropy. The results for Subject 2 are presented in Table 5, which compares the image quality of 20 frames accumulated directly with 20 frames accumulated after registration. This table highlights the improvements in image quality achieved by integrating the GA-CPSO-based registration with the frame accumulation process across all wavelengths. Figure 11 provides a visual comparison for Subject 2 at 620 nm, further confirming the enhancement of overall image quality achieved through registration before accumulation.

The results presented in Table 5 demonstrate significant improvements in image quality when registration is integrated with frame accumulation across all wavelengths. For example, at 620 nm, the EOG increased from (4.2420 × 10^6^) for direct accumulation to (1.6499 × 10^7^) after registration, marking a 3.89-fold improvement. This pattern of enhancement is consistent across all wavelengths, with the 670 nm wavelength showing the highest improvement in EOG, with a 4-fold increase from (4.0418 × 10^6^) to (1.6144 × 10^7^).

Additionally, other metrics such as Brenner gradient, gray level, standard deviation, and entropy saw notable improvements. For the gray level, the highest improvement occurred at 600 nm, increasing from (42.1654) to (49.5544), while SD and entropy metrics also showed substantial gains across all wavelengths, particularly at 670 nm, where SD increased from (25.0007) to (29.9015) and entropy from (6.0492) to (7.6781) in 620 nm. These enhancements collectively indicate that registration is essential for improving not only the sharpness and contrast but also the overall dynamic range and clarity of the images.

Figure 10 visually confirms the quantitative improvements seen in Table 5. The direct accumulation image shows more blurring and less clarity, while the image after registration displays enhanced sharpness and detail, especially in regions affected by motion or misalignment. This visual comparison aligns with the metrics shown in Table 5, validating that the registration step before accumulation plays a crucial role in improving image quality.

Moving to Figure 11 (a result of Subject 2), the quality enhancement brought by registration is further reinforced through a comparison of multiple frames. Here, the plotted metrics demonstrate the quantitative differences between frame accumulation alone and frame accumulation with GA-CPSO-based registration. As more frames are accumulated, the differences between the two methods become increasingly pronounced, with registration consistently improving sharpness, contrast, and detail retention. The increasing gap in metrics such as Brenner gradient and energy of image gradient further highlights the critical role of registration in reducing motion artifacts and enhancing the overall quality of multispectral breast images.

To further extend the comparative analyses, the performance of the GA-CPSO algorithm was also compared with another recently documented image registration technique, the repeated pair image registration and accumulation (RPIRA) based on the gradient descent method [52]. For this comparison, the evaluation metrics including EOG, BGF, and MMD were utilized. Briefly, EOG quantifies the sharpness of image edges, BGF measures image focus, and MMD indicates image contrast. To ensure consistency, we repeated the frame accumulation and registration process using the same methodology described in the RPIRA method, applying both registration algorithms to the same dataset of 20 frames acquired at 670 nm for Subject 1.

An objective comparison of the GA-CPSO and RPIRA methods, based on these metrics, is presented in Table 6. In particular, the EOG values were higher for the GA-CPSO technique than the RPIRA method (10.09 vs. 8.58), indicating sharper edges in images processed with GA-CPSO. Moreover, the GA-CPSO algorithm demonstrated a substantially higher BGF, and consequently better image focus, compared to the RPIRA method (11.35 vs. 1.69). Similarly, the significantly higher MMD metric for the GA-CPSO algorithm revealed superior contrast preservation. It is worth noting that the RPIRA method relies on gradient descent as the registration algorithm, which appears to be less effective than the GA-CPSO approach in preserving image quality metrics. Collectively, these results demonstrate that the GA-CPSO algorithm outperforms the RPIRA method in all evaluated metrics, underscoring its enhanced effectiveness in frame accumulation.

To further expand the comparative analyses, the GA-CPSO technique was compared with the Powell and the TC-NMI algorithms. To enable a one-to-one comparison, three image quality metrics (i.e., EOG, Brenner, and MMD) were computed for GA-CPSO algorithm after accumulating 10 frames of the images acquired at 670 nm. Table 7 presents a summary of these comparative analyses. Overall, the highest performance across all three performance metrics was demonstrated by the GA-CPSO algorithm. Specifically, the GA-CPSO algorithm archived higher EOG metric values, compared to the Powell and TC-NMI algorithms (6.979 × 10^6^, 4.964 × 10^5^, and 1.873 × 10^5^, respectively). This indicates superior edge preservation of the image with the GA-CPSO algorithm. Similarly, the Brenner metric, which evaluates image focus, was 4.908 × 10^6^ for GA-CPSO, compared to 6.329 × 10^5^ for Powell and 2.508 × 10^5^ for TC-NMI methods. Finally, the MMD value for GA-CPSO algorithm was 9.871 × 10^5^, compared to 1.981 × 10^4^ for Powell and 9.472 × 10^3^ for TC-NMI techniques. These results of the objective comparison highlights the superior performance of GA-CPSO method in improving the accumulated image quality, in comparison to the reported techniques. It may be mentioned that, compared to Powell and TC-NMI algorithms, the GA-CPSO algorithm also illustrated better performance in terms of CC, MI, RMSE, and registration time (Table 4). Taken together, the GA-CPSO technique outperformed both the Powell and the TC-NMI algorithms based on a comprehensive set of metrics: CC, MI, RMSE, and registration time to assess registration accuracy and efficiency, and EOG, Brenner, and MMD to evaluate the final image quality after registration and frame accumulation.

### 4.3. The GA-CPSO Registration Method’s Efficiency in Mitigating Ghosting Effects

**Figure 12:**
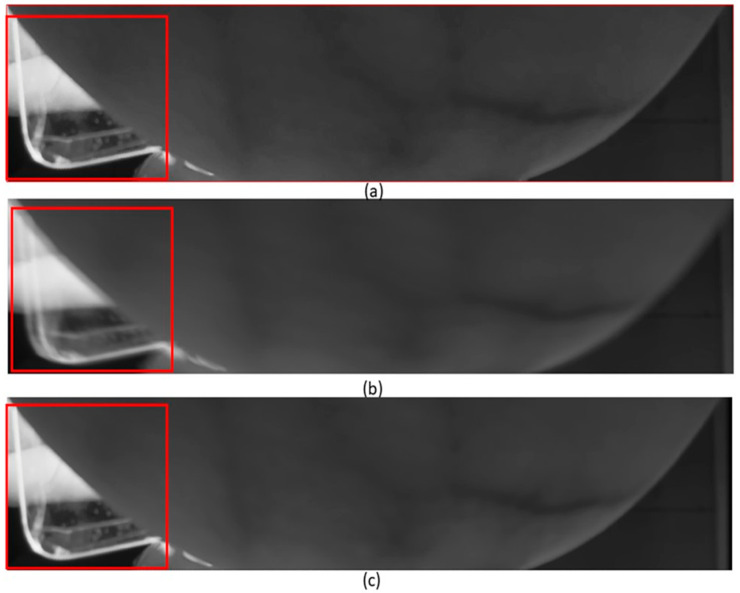
Cropped image showing ghosting effect due to frame accumulation from 670 nm wavelength images: (**a**) original single frame, (**b**) 20 frames direct accumulation, and (**c**) 20 frames accumulation after GA-CPSO registration.

The efficiency of the GA-CPSO registration algorithm in mitigating ghosting effects during frame accumulation was evaluated. Figure 12 visually demonstrates the effectiveness of our approach in enhancing image accuracy and quality, with a specific focus on minimizing ghosting phenomena. This improvement is more pronounced in the areas within the indicators.

Figure 12a depicts a cropped section from the initial frame of a breast image captured at a 670 nm wavelength. Subsequently, Figure 12b portrays the same cropped region from a directly accumulated image of 20 frames at a 670 nm wavelength, revealing noticeable blurring or ghosting effects. These artifacts typically result from slight frame displacements induced by factors such as camera jitter or environmental disturbances during the image capture process. Figure 12c shows the diminished ghosting effect achieved by employing image registration, specifically the GA-CPSO-enhanced algorithm, prior to frame accumulation. The evident reduction in ghosting effects in Figure 12c improved image quality and enhanced accuracy in frame accumulation facilitated by our registration methodology.

Building upon these improvements, the enhancements in metrics such as CC, MI, and RMSE directly translate to improved alignment of multispectral images. The potential of the GA-CPSO algorithm to improve the SNR through accurate registration and frame accumulation reduces the likelihood of false positives or negatives, ensuring more reliable diagnoses. Additionally, by mitigating ghosting and motion artifacts caused by patient movement or respiratory-induced displacements, the hybrid algorithm ensures clearer and more interpretable images, essential for identifying tissue heterogeneity or vascular abnormalities across different wavelengths. The enhanced image quality also supports multispectral imaging’s ability to capture tissue-specific absorption and scattering properties, which are vital for distinguishing between malignant and benign tissues. The algorithm’s robustness enables integration with automated diagnostic systems, streamlining workflows. While focused on breast imaging, its adaptability extends to other modalities like optical coherence tomography and ultrasound, ensuring precise image registration.

Previous studies have reported diverse methods for image registration. For instance, a dual approach of using the TC-NMI method for registering multispectral breast phantom images has been documented [55]. However, this method was computation-intensive and time-consuming, partially due to the grayscale information. Another approach for medical image registration combined a modified MI metric with PSO, successfully incorporating both statistical and spatial information, achieving accurate registration results across CT and MRI images [56]. Moreover, this approach was extended by incorporating the Powell search algorithm to align infrared and visual images [57]. Additionally, a PSO sample consensus (PSO-SC) algorithm was developed for remote sensing image registration to improve the precision of image alignment [38]. The Powell algorithm was applied to optimize the NMI similarity metric to multispectral breast images [58], but this method took too much registration time, lacking time efficiency. The present study combined the global search capabilities of GAs with the fine-tuning attributes of the PSO algorithm in tandem with the SCF. With this novel approach, the speed of image registration and accuracy of frame alignment were significantly improved in multispectral breast images.

## 5. Comparison with Previous Studies

Frame displacement in sequential images acquired at different time points compromise accuracy of frame accumulation and overall image quality. A number of algorithms have been designed to eliminate variance in matching of different image frames [59]. For multispectral breast images, the registration accuracy was enhanced by using the gradient descent algorithm and summing the pixel values of multi-wavelength signals at each pixel point prior to image registration [60]. This technique resulted in a CC of ~0.668, MI of ~2.294, and RMSE of ~7.0416 for breast images captured at 620 nm [60]. A strategy integrating frame accumulation and image registration mediated by NMI was used to improve the accuracy and quality of image registration for breast transmission images [54]. Moreover, this hybrid approach diminished the image jitter-related issues and amplified operation speed [54]. The EOG, Brenner gradient, and MMD reported for breast images at 600 nm were 1.5956 × 10^8^, 1.9440 × 10^8^, and 3.8986 × 10^7^, respectively [54]. In another novel approach, multispectral breast images were registered and accumulated in pairs. Briefly, from the acquired sequence, registration (based on the RPIRA method) of the first pair of multispectral breast images was followed by the registration and accumulation of next pair of images. Iteration of this process continued until all image frames of the sequence were processed. The final image thus obtained demonstrated improved accuracy. This algorithm resulted in a CC of ~0.979 and an MI of ~3.600 for breast images acquired at 600 nm [52]. For comparison, the values EOG, Brenner gradient, and MMD for the GA-CPSO technique used in this study were 1.009 × 10^7^, 1.135 × 10^8^, and 1.423 × 10^6^, respectively.

To detect and correct for patient movement during imaging, a 3D rigid transformation was decomposed in two orthogonal 2D projections (i.e., in-plane transformations and out-of-plane rotations) with the use of an approximate geometric relationship. These 2D projections were then combined and converted back to a 3D rigid transformation, using a 2D–3D geometric transformation. This 2D–3D image registration algorithm claimed to provide a sub-millimeter accuracy for the frame accumulation [61]. This approach demonstrated an error (i.e., frame displacement) of 0.38, 0.21, and 0.76 mm in the directions of anterior/posterior (x-axis), left/right (y-axis), and superior/inferior (z-axis), respectively [61]. Moreover, the Powell method also offers a simple and fast algorithm for image registration. This algorithm has been integrated with other contemporary methods to increase the accuracy of medical image registration. For instance, the Powell algorithm in tandem with histogram equalization of images showed improved accuracy for frame accumulation, in terms of SNR, MSE, time-efficiency, and CC [58]. This technique resulted in an MI of ~1.906, normalized MI of ~1.255, and NCC of ~0.920 for breast images [58]. Likewise, a dual approach with Powell and slime mold algorithms enabled improved registration accuracy by homogenizing the local maxima [62].

## 6. Conclusions

This study demonstrated a substantial enhancement in the quality of multispectral transmission imaging of breast tissue through the utilization of the GA-CPSO algorithm for image registration before frame accumulation. By effectively optimizing the GA-CPSO approach, it overcomes challenges such as camera jitter and respiratory-induced frame displacement, significantly enhancing image quality. The hybrid method achieves high accuracy (~99.93%). The registration method’s efficiency was evaluated using multiple metrics, including registration accuracy, correlation coefficient (CC), mutual information (MI), and root mean square error (RMSE), and was compared with two other methods, demonstrating superior performance in both accuracy and time efficiency. Consequently, this method not only improves CPSO registration accuracy but also enhances the effectiveness of frame accumulation technology, showing superior performance in reducing artifacts and noise, and improving contrast and pixel intensity distribution of accumulated image. The quality of the images was further evaluated using metrics such as the energy of image gradient (EOG), Brenner gradient, gray level (GL), standard deviation (SD), and entropy, all of which showed marked improvements after the integration of registration with frame accumulation. The integration of our registration method with frame accumulation significantly enhances the signal-to-noise ratio (SNR) and overall image quality of multispectral images, providing a strong foundation for further processing techniques such as segmentation and the detection of breast tissue anomalies. The observed improvements in image quality suggest the potential of the GA-CPSO framework for complementing the existing imaging techniques in early breast cancer detection. However, further studies with standardized clinical imaging setups are necessary to validate its diagnostic utility.

## Figures and Tables

**Table 1:** Registration speed enhancement with squared constriction factor in CPSO algorithm.

Image Path		Iteration Number		Best Value		Registration Time (s)	
	Wavelength (nm)	CPSO with CCF	CPSO with SCF	CPSO with CCF	CPSO with SCF	CPSO with CCF	CPSO with SCF
1	600	83	43	1.36	1.34	411.69	192.56
2	600	93	44	3.75	3.76	370.09	188.09
3	620	94	36	2.61	2.60	373.20	190.19
4	620	64	43	6.18	6.18	364.11	187.81
5	670	95	46	6.08	6.08	362.58	180.27
6	670	96	45	17.34	17.34	370.33	181.08
7	760	82	32	7.09	7.08	376.42	185.27
8	760	91	37	22.63	22.62	382.70	192.76

CPSO: constriction factor-based particle swarm optimization, CCF: conventional constriction factor, SCF: squared constriction factor.

**Table 2:** Accuracy comparison of CPSO alone and the hybrid GA-CPSO algorithm for moving image registration (Subject 1).

Wavelength Parameters	600 nm (20, 20, 0.5)		620 nm (30, 25, −5)		670 nm (50, −50, 10)		760 nm (10, 10, −15)	
	CPSO	GA-CPSO	CPSO	GA-CPSO	CPSO	GA-CPSO	CPSO	GA-CPSO
x displacement	19.025	20.009	29.415	30.055	49.990	50.027	09.599	09.959
y displacement	19.600	19.966	24.541	24.990	−50.689	−49.955	10.059	10.089
Angle θ displacement	00.505	00.500	−05.170	−05.000	10.180	10.004	−15.032	−15.000
Accuracy (%)	97.375	99.928	97.605	99.925	98.934	99.939	98.396	99.566

**Table 3:** Accuracy comparison of CPSO alone and hybrid GA-CPSO algorithm for moving image registration (Subject 2).

Wavelength Parameters	600 nm (05, 05, 0.2)		620 nm (50, 45, −10)		670 nm (25, −20, 05)		760 nm (−80, 60, 15)	
	CPSO	GA-CPSO	CPSO	GA-CPSO	CPSO	GA-CPSO	CPSO	GA-CPSO
x displacement	05.016	05.008	50.606	49.891	24.596	25.059	−79.299	−79.878
y displacement	05.239	05.002	44.898	45.013	−20.193	−20.090	60.703	60.025
Angle θ displacement	00.201	00.200	−10.160	−10.000	05.120	05.000	15.120	15.000
Accuracy (%)	98.133	99.933	98.987	99.918	98.340	99.771	99.051	99.935

**Table 4:** Comparison of GA-CPSO algorithm with other methods.

Evaluation Metric	GA-CPSO Algorithm	Powell Algorithm [54]	TC-NMI [55]
600 nm			
CC	0.9950	0.9702	0.9785
MI	4.2474	3.2390	3.9065
RMSE	1.5129	3.6545	6.0218
Registration time (s)	406.57	3638.60	3513.10
620 nm			
CC	0.9982	0.9531	0.9510
MI	3.9921	1.3533	3.0554
RMSE	1.6419	9.2852	6.9321
Registration time (s)	388.80	3670.60	2540.83
670 nm			
CC	0.9896	0.9669	0.9528
MI	4.4218	4.0118	3.8401
RMSE	1.1218	2.8039	15.1538
Registration time (s)	397.15	4864.52	2691.00
760 nm			
CC	0.9963	0.9268	0.9697
MI	4.3894	3.1888	3.9803
RMSE	2.9488	10.7126	8.7365
Registration time (s)	397.88	5864.50	9423.78

TC-NMI: terrace compression with normalized mutual information, CC: correlation coefficient, MI: mutual information, RMSE: root mean square error.

**Table 5:** Comparison of image quality for direct accumulation and GA-CPSO algorithm-based registration plus accumulation (Subject 2).

Final Image	EOG	Brenner	GL	SD	Entropy
600 nm					
Direct accumulation	2.6854 × 10^6^	3.9852 × 10^6^	42.1654	21.9603	5.2871
Registration + accumulation	9.4465 × 10^6^	9.6038 × 10^6^	49.5544	22.8078	6.0456
620 nm					
Direct accumulation	4.2420 × 10^6^	9.5382 × 10^6^	45.0301	33.4953	6.0492
Registration + accumulation	1.6499 × 10^7^	3.3134 × 10^7^	47.3931	36.6195	7.6781
670 nm					
Direct accumulation	4.0418 × 10^6^	9.8232 × 10^6^	53.6046	25.0007	6.0260
Registration + accumulation	1.6144 × 10^7^	3.5610 × 10^7^	56.3670	29.9015	7.4870
760 nm					
Direct accumulation	2.5743 × 10^6^	4.8203 × 10^6^	52.7060	37.0349	6.8005
Registration + accumulation	5.7916 × 10^6^	6.7017 × 10^6^	57.1003	40.5402	7.4568

**Table 6:** Comparison of GA-CPSO algorithm with the RPIRA method (based on gradient descent).

Metrics	Proposed GA-CPSO	Gradient Descent [52]
EOG (×10^6^)	10.09	8.58
Branner (×10^7^)	11.35	1.69
MMD (×10^5^)	14.23	8.44

**Table 7:** Comparison of GA-CPSO, Powell, and TCM-NMI methods after accumulating 10 frames.

Metrics	Proposed GA-CPSO	Powell Algorithm [54]	TC-NMI [55]
EOG (×10^5^)	69.79	4.96	1.87
Branner (×10^5^)	49.08	6.33	2.51
MMD (×10^4^)	98.71	1.98	0.95

## Data Availability

Data supporting the findings of this study are contained within the article.
